# Diagnostic re-evaluation of non-odontogenic orofacial pain after treatment failure: a retrospective case series

**DOI:** 10.22514/jofph.2026.039

**Published:** 2026-05-12

**Authors:** Hao Chih Chuang, Hisashi Sato, Kosei Kubota, Wataru Kobayashi

**Affiliations:** ^1^Department of Dentistry and Oral Surgery, Tsugaru General Hospital, 037-0074 Aomori, Japan; ^2^Department of Oral and Maxillofacial Surgery, Hirosaki University Graduate School of Medicine, 036-8563 Aomori, Japan

**Keywords:** Orofacial pain, Persistent dentoalveolar pain disorder, Trigeminal neuralgia, Differential diagnosis, Non-odontogenic pain

## Abstract

**Background**: Orofacial pain often arises from overlapping odontogenic and non-odontogenic causes, which can lead to misdiagnosis and unnecessary irreversible procedures. Practical guidance on diagnostic re-evaluation in treatment-refractory cases with negative dental findings remain limited. **Methods**: This retrospective case series describes six illustrative patients (one man and five women; median age, 74.5 years) selected from referrals for suspected non-odontogenic orofacial pain, based on diagnostic difficulty, treatment refractoriness, and objective negative dental findings. Two oral and maxillofacial surgeons independently re-evaluated each case using the International Classification of Orofacial Pain, the 3rd edition of the International Classification of Headache Disorders, and the Diagnostic Criteria for Temporomandibular Disorders. **Results**: The final or most strongly suspected diagnoses included persistent idiopathic dentoalveolar pain, lingual nerve injury, trigeminal neuralgia, trigeminal autonomic cephalalgia, and myofascial pain. The median diagnostic delay was 17 months (interquartile range, 7–96). Initial empiric dental management (*e.g.*, antibiotics and/or nonsteroidal anti-inflammatory drugs (NSAIDs)) was insufficient in all cases. Pain intensity improved from a median numerical rating scale score of 8 at baseline to 0–2 at final observation. Across cases, common diagnostic pitfalls included premature attribution to a single cause, overreliance on imaging or prior medication history, and insufficient musculoskeletal or neurological examination. One or more clinical features prompting diagnostic re-evaluation were observed in each case, including persistent pain with objective negative dental findings, autonomic signs, or incongruent treatment response. **Conclusions**: In cases of treatment-refractory orofacial pain with objective negative dental findings, suspending further irreversible interventions and conducting systematic diagnostic re-evaluation may help reduce diagnostic delays and avoid unnecessary procedures.

## 1. Introduction

Orofacial pain is frequently encountered in clinical practice [1] and can broadly result from odontogenic and non-odontogenic causes. Non-odontogenic orofacial pain is clearly defined in the International Classification of Orofacial Pain (ICOP) and the International Classification of Headache Disorders, 3rd edition (ICHD-3); it encompasses diverse conditions, including musculoskeletal and neurological disorders of the stomatognathic system and primary headaches [2, 3, 4]. Clinically, non-odontogenic orofacial pain comprises several heterogeneous categories, including neuropathic pain (*e.g.*, persistent dentoalveolar pain and post-traumatic trigeminal neuropathy), temporomandibular disorders, myofascial pain, neurovascular headache, and pain secondary to systemic or central nervous system disorders [5, 6]. These conditions frequently present with overlapping clinical features and limited objective findings, making differentiation from odontogenic pain particularly challenging in routine dental practice. This condition can present as toothache-like “referred” pain and requires careful differentiation from odontogenic pain. Inadequate differentiation can result in persistent symptoms after irreversible dental procedures, leading to diagnostic delays and unnecessary intervention [7, 8, 9, 10].

In clinical settings, overemphasis on imaging, prior treatment response, insufficient musculoskeletal or neurological examination, and premature attribution to a single cause increase the risk of misdiagnosis [7, 11]. In particular, when radiographic findings are negative or nonspecific, diagnostic reasoning may remain confined to an odontogenic framework, despite persistent pain that is disproportionate to clinical findings. Accordingly, current guidelines emphasize systematic assessment, including history taking, provocation tests, musculoskeletal and neurological findings, identification of headache-associated signs, and staged differential diagnosis with periodic re-evaluation [2, 3, 12]. Such an approach requires familiarity with diagnostic criteria and clinical judgment to determine when initial working diagnoses should be reconsidered in the absence of a treatment response. From an epidemiological perspective, chronic orofacial pain is more common in women than in men [1, 13].

The present study adopts an ICOP terminology and uses persistent idiopathic dentoalveolar pain (PIDP) as the core concept. Given its entrenched usage in Japanese dental practice and literature, the term persistent dentoalveolar pain disorder (PDAP) is presented in parallel where contextually appropriate. We did not use the obsolete term atypical odontalgia given its ambiguous definition.

In rapidly aging societies such as Japan, where over 28% of the population is aged 65 years or older [14], the clinical burden of chronic or treatment-refractory orofacial pain in older adults is projected to increase, making non-odontogenic causes of orofacial pain an increasingly important diagnostic consideration. Older patients often present with multimorbidity, polypharmacy, and atypical pain presentations, further complicating diagnostic evaluation in general dental practice.

However, the accurate diagnosis and appropriate management of non-odontogenic orofacial pain are often delayed in routine dental practice [8, 9]. Consequently, pain that cannot be readily explained by odontogenic pathology is commonly managed within a narrow dental framework, often leading to the repeated performance of irreversible procedures, such as tooth extraction, endodontic retreatment, or surgical curettage [7, 8, 9, 10]. This tendency towards overreliance on odontogenic explanations may delay the recognition of non-odontogenic pain mechanisms, such as neuropathic, musculoskeletal, and neurovascular disorders [7, 15, 16]. Consequently, patients may experience prolonged diagnostic delay, unnecessary invasive treatment, and progression to chronic pain states [7, 8, 15].

Despite the growing clinical relevance of this problem, studies describing how systematic diagnostic re-evaluation can alter clinical trajectories in patients with persistent orofacial pain and negative dental findings remain limited [8, 9]. Practical frameworks for identifying clinical signals or decision points that indicate when to suspend further dental intervention and reassess alternative diagnoses, particularly in the oral and maxillofacial surgery setting, are currently lacking [7, 8]. This retrospective case series study aimed to characterize the clinical features and diagnostic processes of patients with treatment-refractory orofacial pain and negative dental findings, as well as to highlight the clinical value of a structured “stop-and-re-evaluate” approach in this increasingly common scenario.

## 2. Materials and methods

### 2.1 Study design and population

This was a retrospective case series. Among the 1824 new patients who presented to the Department of Dentistry and Oral and Maxillofacial Surgery of a regional general hospital between April 2023 and April 2025, 54 had a chief complaint of orofacial pain and were referred from community dental clinics for further evaluation and management due to suspected non-odontogenic orofacial pain.

#### 2.1.1 Inclusion criteria

Cases were considered eligible if they met both of the following criteria:

(1) Difficulty establishing a definitive odontogenic or non-odontogenic diagnosis at the first visit and/or after initial treatment. “Diagnostic difficulty” was defined as fulfilling all the following criteria:

(a) no evident odontogenic pathology on imaging,

(b) no signs of acute intraoral inflammation, and

(c) inability to reproduce the patient’s pain with percussion, occlusion, or thermal testing.

(2) Persistence or relapse of pain despite multiple treatment interventions. “Persistence” was defined as one of the following:

(a) after two or more dental interventions (*e.g.*, pulpectomy, extraction, antibiotics, NSAIDs), each lasting ≥2 weeks, the numerical rating scale (NRS) score did not improve by ≥2 points from baseline; or

(b) after ≥4 weeks of appropriate-dose initial pharmacotherapy, the NRS score did not improve by ≥2 points from baseline or showed recurrent relapses after initial improvement.

#### 2.1.2 Exclusion criteria

Cases were excluded if they met any of the following criteria:

(1) identification of a definitive odontogenic pathology (*e.g.*, acute pulpitis, vertical root fracture, or periodontal abscess) that fully explain the symptoms;

(2) rapid symptom resolution following initial standard dental treatment or first-line pharmacotherapy;

(3) presence of pain attributable to the normal postoperative healing process following recent dental procedures;

(4) diagnosis of primary headache disorders (*e.g.*, migraine or tension-type headache) requiring referral to the neurology department at the initial visit;

(5) pain clearly attributable to systemic diseases (*e.g.*, metastatic malignancies or autoimmune disorders) or central nervous system pathologies identified during screening; and

(6) incomplete clinical records preventing adequate data extraction or follow-up assessment.

### 2.2 Standardized provocation tests

Percussion testing involved vertical and lateral taps with a mouth mirror handle, occlusal testing involved a cotton roll and natural occlusion, and thermal testing used ethyl chloride spray and warm water (40 °C). Failure to reproduce the patient’s usual pain with any provocation was defined as “provocation negative”.

Pain intensity was assessed using an 11-point NRS (0–10), with clinically meaningful change defined as ≥2-point improvement.

### 2.3 Neuromuscular and chairside sensory examination

All patients underwent systematic musculoskeletal and neurosensory evaluations as part of the standardized clinical re-evaluation protocol. Musculoskeletal examination included the following: (1) Bilateral palpation of the masticatory muscles (masseter and temporalis) to assess tenderness, muscle stiffness, and trigger points; (2) Bilateral palpation of the temporomandibular joints (TMJs), including the lateral pole and posterior attachment areas; (3) Trigger point provocation testing using the application of standardized moderate digital pressure for several seconds to determine whether the patient’s familiar pain could be reproduced; (4) Assessment of side-to-side differences in muscle tenderness and TMJ findings, involving neurosensory examination conducted using chairside sensory testing, rather than formal instrument-based quantitative sensory testing (QST).

Specifically, neurosensory examination comprised the following assessments: (i) Light-touch detection involving qualitative assessment using a cotton swab applied bilaterally to the region corresponding to the trigeminal nerve distributions; (ii) Touch-pressure detection, evaluated using Semmes–Weinstein monofilaments when somatosensory abnormality was suspected; (iii) Mechanical pain sensitivity, examined using a sterile dental explorer (pinprick test) with gentle stimulation across the affected and contralateral sides. All sensory assessments were performed using side-to-side comparison to identify asymmetry, including hypoesthesia (reduced sensation), hyperesthesia (increased sensation), or allodynia (pain from non-noxious stimuli). These chairside assessments were conducted to screen clinically relevant somatosensory abnormalities, as well as to guide diagnostic reclassification in conjunction with clinical history, provocation test findings, and imaging results. Standardized computerized QST equipment was not available in the clinical setting. Thermal provocation testing (ethyl chloride spray for cold, warm water at approximately 40 °C) was conducted as part of dental provocation tests (described in Section 2.2), rather than as systematic thermal threshold assessment.

### 2.4 Diagnostic workflow and case sorting

Based on a retrospective review of referred cases with unresolved orofacial pain, we organized the clinical decision points and re-evaluation steps into a conceptual diagnostic workflow. This framework was not applied as a predefined protocol at the time of initial diagnosis, but represents a *post-hoc* synthesis of the clinical reasoning processes used across cases. The resulting diagnostic and re-evaluation framework is illustrated in Fig. 1.

**Fig. 1.** Algorithm for diagnosis and re-evaluation of non-odontogenic orofacial pain. Initial assessment includes systematic history (pain characteristics, duration, triggers, associated symptoms), clinical examination (intraoral exam, imaging, provocation tests), and musculoskeletal/neurological evaluation. Red flags: (1) persistent pain + objective negatives; (2) ipsilateral autonomic signs (nasal congestion, conjunctival injection, tearing); (3) nonresponse to usual dental treatment. When present, pause irreversible procedures and proceed with differential diagnosis per ICOP/ICHD-3/DC/TMD; refer classic cases (paroxysmal electric pain; TACs signs) to appropriate specialists. If red flags are absent and clear odontogenic pathology is identified, provide standard dental care, reassess NRS every 2–4 weeks, and return to re-evaluation if improvement is insufficient. ICOP: International Classification of Orofacial Pain; ICHD-3: International Classification of Headache Disorders, 3rd edition; DC/TMD: Diagnostic Criteria for Temporomandibular Disorders; NSAIDs: nonsteroidal anti-inflammatory drugs; TACs: trigeminal autonomic cephalalgias; PIDP: persistent idiopathic dentoalveolar pain; PDAP: persistent dentoalveolar pain disorder; NRS: numerical rating scale; RCT: Root Canal Treatment; TN: Trigeminal Neuralgia; TCA: Tricyclic Antidepressant; PT: Physical Therapy.

Using this workflow, cases were triaged and re-evaluated as follows: Among the 54 referred cases, 50 were confirmed to have non-odontogenic pain, while 4 showed odontogenic pathology and were returned to the referring clinics. Of the 50 non-odontogenic cases, 41 showed a rapid response to first-line pharmacotherapy or management protocols specific to their diagnoses (*e.g.*, carbamazepine for typical trigeminal neuralgia, splint therapy for temporomandibular disorders) in our department. In three patients, primary headache was strongly suspected at the initial visit, and they were directly referred to the neurology department. Six cases showed difficulty with definitive diagnosis or symptom control and were designated as the analytical cohort. Among these, one case (Case 4) was referred to the neurology department after ineffective initial treatment and was subsequently diagnosed with trigeminal autonomic cephalalgias (TACs). In total, four cases were referred to the neurology department.

### 2.5 Diagnostic procedure

Final diagnoses were assigned independently by two oral and maxillofacial surgeons (board-certified specialists with 30 years of experience and board-certified dentists with 6 years of experience) based on the ICOP, ICHD-3, and Diagnostic Criteria for Temporomandibular Disorders (DC/TMD) operational criteria. For PIDP, the ICOP criteria were applied as follows: (1) pain persisting or recurring for >3 months, (2) pain located in the dentoalveolar region for >2 h per day, (3) no clear odontogenic or other pathology, and (4) lack of response to usual dental treatments. Any discrepancies were resolved by consensus.

### 2.6 Data collection

We extracted the following data from the medical records: demographics, medical history, clinical findings (site/quality of pain and associated symptoms), imaging findings, neurological/musculoskeletal findings, treatments, and treatment responses. Follow-up data were collected until August 2025, with some patients remaining under observation at that time. Medications were recorded using generic names. Regarding kampo co-therapy, we recorded its presence/absence, formula names, and treatment response. In some cases, kampo served as an adjunctive background therapy and was excluded from the primary efficacy analyses. Tooth numbering was performed using the FDI two-digit tooth numbering system (FDI system). Diagnostic delay (months) was defined as the period from symptom onset to final diagnosis (or provisional diagnosis) at our department. Missing items in referral letters/records were labeled “NA”. Table 1 summarizes the evaluation items (variable definitions and denominators in the footnotes).

**Table 1.** Evaluation items and operational definitions.

Category	Item	Definition/Measurement Criteria	Data Source
Background Information	Age/Sex/Medical History	Based on records at the initial visit	Medical chart
Pain-related Information	Site/Characteristics/Duration	Assessment of subjective symptoms	Referral letter/Medical chart
Imaging Findings	Odontogenic lesion/Bone change	Evaluation using panoramic radiograph or CBCT	Imaging
Provocation Tests	Pain reproduction by percussion, occlusion, or thermal stimulation	Positive if reproduced, negative if not reproduced	Clinical findings
Treatment History	Pulpectomy, tooth extraction, antibiotics, NSAIDs, *etc*.	Number and duration of treatments recorded	Referral letter/Medical chart
Pain Intensity	Numerical Rating Scale (NRS, 0–10)	Improvement of ≥2 points defined as clinically significant	Chart record
Neuromuscular Findings	Muscle tenderness, sensory disturbance, limitation of mouth opening, *etc.*	Positive/Negative	Clinical findings
Autonomic Symptoms	Nasal obstruction, conjunctival injection, lacrimation, *etc.*	Present/Absent	Interview/Visual inspection
Diagnostic Delay	Duration from symptom onset to final diagnosis	Measured in months	Chart/Patient report

*NSAIDs: nonsteroidal anti-inflammatory drugs; NRS: numerical rating scale; CBCT: Cone-beam computed tomography.*

To facilitate systematic diagnostic re-evaluation and enable transparent cross-case comparison, a structured data extraction framework (Table 1) was applied. Table 1 summarizes the core clinical variables and operational definitions used in this study, organized into nine categories: background information, pain-related information, imaging findings, provocation tests, treatment history, pain intensity, neuromuscular findings, autonomic symptoms, and diagnostic delay. This standardized approach ensured consistent evaluation across all cases, and provided the basis for identifying recurring diagnostic pitfalls.

### 2.7 Identification of misdiagnosis factors

Based on a qualitative review of the six cases, factors contributing to misdiagnosis were identified and categorized into four domains through an author-derived clinical synthesis. This process was informed by established concepts in the diagnostic error literature, including cognitive biases (*e.g.*, anchoring and premature closure), incomplete clinical assessment, and contextual factors [7, 11, 17, 18]. This framework was exploratory in nature and was not intended to represent a previously validated classification system.

### 2.8 Statistical analysis

Only descriptive statistics were used. All statistical analyses were performed using spreadsheet software (Microsoft Excel, Microsoft 365; Microsoft Corp., Redmond, WA, USA).

### 2.9 Case descriptions

An overview of the clinical courses and final diagnoses for the six cases is provided in Table 2.

**Table 2.** Clinical characteristics and clinical course of the six cases.

Case	Age/Sex	Chief Complaint/Site	Initial Diagnosis & Treatment	Final Diagnosis	Treatment at Our Department	NRS Change	Diagnostic Duration (mon)
Case 1	78/F	Persistent pain in the right upper anterior tooth region	Pulpitis → RCT, extraction (FDI 13). Antibiotics and NSAIDs ineffective.	Persistent idiopathic dentoalveolar pain (PIDP/PDAP, provisional)	Mirogabalin, Yokukansan, Kamishoyosan	8 → 0	3
Case 2	58/F	Burning-stinging dysesthesia at the right base of the tongue	Post-extraction state (FDI 47, 48). Interpreted as chemotherapy side effect.	Traumatic lingual neuropathy (Tentative diagnosis)	Mirogabalin, Amitriptyline	8 → 1	24
Case 3	90/F	Paroxysmal electric shock-like pain in the right lower molar region	Extraction → Re-curettage → Alveoloplasty. MRONJ stage 0 suspected. Antibiotics and NSAIDs ineffective.	Trigeminal neuralgia	Carbamazepine	8 → 0	10
Case 4	74/M	Pain in the region corresponding to the maxillary anterior teeth	Accompanied by nasal congestion and conjunctival injection. Odontogenic findings negative.	Trigeminal autonomic cephalalgia (TACs)	Referral to Neurology → Pregabalin	8 → 3	1.4
Case 5	65/F	Pain in the left maxillary and mandibular regions	Pain persisted despite multiple pulpectomies, extractions, and apicoectomies. Exacerbated in supine position.	Mixed pain (neuropathic + myofascial pain) (Tentative diagnosis)	Carbamazepine, Celecoxib	8 → 1–2	72
Case 6	75/F	Persistent pain in the left upper and right lower gingival regions	Trigeminal neuralgia suspected → Carbamazepine ineffective. MRI showed neurovascular contact.	Chronic myofascial pain (latent trigger points)	Celecoxib, Eperisone, Yokukansan, Sokeikakketsuto	1–2 → 0	120

*F: Female; M: Male; NSAIDs: nonsteroidal anti-inflammatory drugs; NRS: numerical rating scale; PIDP: persistent idiopathic dentoalveolar pain; PDAP: persistent dentoalveolar pain disorder; RCT: root canal treatment; FDI: FDI two-digit tooth numbering system; MRONJ: medication-related osteonecrosis of the jaw; MRI: magnetic resonance imaging.*

#### 2.9.1 Case 1

Patient: A 78-year-old woman with a history of hypertension.

Chief complaint/course: On January 2025, the patient presented with persistent pain in the right maxillary central/lateral region (NRS score = 8; diurnal fluctuation; unrelated to mastication). In February, she was diagnosed with pulpitis of the right maxillary canine (FDI 13) in another clinic and underwent root canal treatment and extraction without improvement. NSAIDs and antibiotics were ineffective.

Initial visit (April; 3 months after onset): The extraction socket had healed; no odontogenic abnormality was noted on imaging; chewing or occlusion did not provoke pain.

Interventions/outcome: Mirogabalin 5 mg/day plus *yokukansan* reduced the NRS score from 8 to 2–3; further, addition of *kamishoyosan* resulted in an NRS score of 0. Remission was maintained after discontinuation of mirogabalin with a good course.

Final diagnosis: Persistent idiopathic dentoalveolar pain (PIDP/PDAP, provisional).

#### 2.9.2 Case 2 (post-extraction, FDI 47)

Patient: A 58-year-old woman with a history of breast cancer.

Course: Burning/stinging dysesthesia of the right tongue root emerged immediately after extraction of the right mandibular second molar (FDI 47) in January 2023. Chemotherapy was initiated at approximately the same time, with the symptoms being attributed to adverse effects for 18 months. The right mandibular third molar (FDI 48) was extracted in July 2023; however, the symptoms persisted. After chemotherapy ended, the symptoms remained unchanged for 6 months, which prompted evaluation at our clinic (~24 months after onset).

Initial findings: Unremarkable imaging findings; symptoms localized to the right side of the tongue; negative dental findings.

Management/outcome: A traumatic lingual nerve injury was suspected. Mirogabalin 10 mg/day plus amitriptyline reduced the NRS score from 8 to 1, as well as improved sleep and mood.

Clinical interpretation: Chemotherapy-induced peripheral neuropathy is typically bilateral and length-dependent in the extremities, with unilateral lingual localization being an atypical finding. Temporal proximity to extraction, localization, and treatment response suggested a traumatic mechanism; however, the involvement of chemotherapy could not be fully ruled out.

Final diagnosis: Traumatic lingual nerve injury (provisional; see Discussion).

#### 2.9.3 Case 3

Patient: A 90-year-old woman with a history of hypertension, subarachnoid hemorrhage, and osteoporosis (alendronate until late 2021).

Course: The patient underwent extraction for right mandibular molar pain in February 2023. Persistent pain led to re-curettage in May without improvement. Given the history of bisphosphonate treatment, osteomyelitis was suspected; however, antibiotic/NSAID prescriptions did not yield any benefit. No apparent bone resorption was observed on radiographs. In October 2023, she was diagnosed with medication-related osteonecrosis of the jaw (MRONJ) stage 0. However, the pain persisted following repeat alveoloplasty.

Initial visit (December 2023; ~10 months after extraction): Paroxysmal electric-shock pain in the right mandibular gingiva was triggered by contact/mastication, which lasted several seconds. There were normal imaging findings.

Management/outcome: The patient was diagnosed with trigeminal neuralgia according to the ICHD-3 criteria. Carbamazepine 100 mg/day abolished the pain within days, with no relapse until July 2025.

Final diagnosis: Trigeminal neuralgia.

#### 2.9.4 Case 4 (edentulous maxilla)

Patient: A 74-year-old man presented with an edentulous maxilla.

Course: Pain in the anterior maxilla was accompanied by nasal congestion and conjunctival injection. There was no odontogenic disease.

Initial approach/referral: Denture misfit was suspected, but adjustments did not yield improvements. Autonomic signs raised suspicion for TACs. Accordingly, he was referred to the neurology department 42 days after initial visit.

Specialty course: TACs (unspecified subtype) was confirmed according to the ICHD-3 criteria. Pregabalin (50 mg/day) therapy was initiated, which stabilized the patient with ongoing neurological management.

Final diagnosis: TACs (unspecified subtype).

#### 2.9.5 Case 5 (diagnosis pending)

Patient: A 65-year-old woman with a history of hypertension.

Course: Since 2019, the patient had experienced pain in the left maxilla and mandible. The pain persisted despite multiple pulpectomies and extractions. At the first visit in July 2020, root-end surgery for fenestration was performed; however, occlusal pain persisted. PDAP was suspected and a tricyclic antidepressant was initiated, which yielded transient relief (NRS 1–2). In August 2021, masseter/temporal tenderness and a limited opening appeared, which was suggestive of DC/TMD type I. The patient discontinued follow-up in September 2021 despite having mild residual symptoms. In April 2025, mandibular pain recurred (NRS score = 8; worse in the supine position). Carbamazepine 200 mg/day plus celecoxib 400 mg/day improved the NRS score to 1–2. As of August 2025, the symptoms have remained stable.

Interpretation: The diagnosis and symptoms evolved over time with differential treatment responses, which suggested the coexistence of neuropathic and myofascial mechanisms.

Final diagnosis: Mixed chronic orofacial pain (neuropathic + myofascial, provisional).

#### 2.9.6 Case 6

Patient: A 75-year-old woman with a history of Sjogren syndrome.

Course: The patient presented with persistent pain in the left maxillary and right mandibular gingiva since 2015. She was referred after a negative otolaryngological evaluation. Trigeminal neuralgia was suspected and carbamazepine was administered without any benefit. Imaging revealed apical disease in the left maxillary second molar (FDI 27), which was extracted, leading to improvement in the left odontogenic pain. Right-sided pain persisted; neurosurgical Magnetic resonance imaging (MRI) showed vascular contact (right superior cerebellar artery) with the trigeminal nerve; however, the ICHD-3 criteria of typical paroxysms and trigger zones were absent. Similar right-sided pain recurred in 2020 and 2022. Upon re-evaluation in March 2025, she had persistent prickling pain (NRS 1–2) in the right upper and lower jaws, which worsened with chewing. There were no changes in the repeat MRI findings.

Findings: No apical lesions or percussion tenderness was observed in the right mandibular first or second molars (FDI 46/47). Masseter/temporal tenderness was present; however, it did not fully reproduce the usual pain. The patient reported relief with self-administered NSAIDs.

Management/outcome: Celecoxib, eperisone, and *yokukansan* eliminated the pain within 2 days (NRS score, 0). Treatment was stopped owing to adverse effects (marked weakness); however, the pain did not recur. Residual tenderness was managed with self-massage/stretching and addition of *Sokyō-kakketsu-tō*. Tenderness improved at 2 weeks, and no relapse has been noted for 3 months. The patient was discharged.

Final diagnosis: Chronic myofascial pain.

## 3. Results

The main factors contributing to misdiagnosis across the six analytical cases are summarized in Table 3.

**Table 3.** Misdiagnosis factors and red flag patterns identified in each case.

Case	① Over-reliance on Imaging/Medication History	② Missed Autonomic Symptoms	③ Insufficient Neuromuscular Examination	④ Premature Attribution to a Single Etiology	Red Flag 1 (Persistent Pain + Negative Provocation Tests)	Red Flag 2 (Autonomic Symptoms)	Red Flag 3 (Inconsistent Treatment Response)
1	√	–	–	√	√	–	√
2	–	–	√	√	√	–	√
3	√	–	√	–	√	–	√
4	–	√	–	√	–	√	√
5	–	–	√	√	√	–	√
6	√	–	√	–	√	–	√
Frequency	3/6 (50%)	1/6 (17%)	4/6 (67%)	4/6 (67%)	5/6 (83%)	1/6 (17%)	6/6 (100%)

*√: Applicable; –: Not applicable; NA: Insufficient information or not assessable. Definitions: Red Flag 1: No radiographic evidence of any odontogenic lesion and provocation tests (percussion, occlusion, thermal stimuli) are negative. Red Flag 2: Presence of ipsilateral autonomic symptoms (nasal congestion, conjunctival injection, lacrimation). Red Flag 3: Incongruent treatment response—insufficient response to usual dental management and/or atypical response patterns suggesting an alternative or mixed pain mechanism. Assessment Method: Evaluations were performed independently by two oral and maxillofacial surgeons, and final determinations were reached by consensus.*

We analyzed six cases that failed to improve despite initial diagnosis and treatment. In cases 1, 2, 3, and 5, care was initiated for reported toothache, which was initially judged to be odontogenic/inflammatory, leading to irreversible procedures (pulpectomy/extraction). In Cases 4 and 6, concomitant pain sites prompted avoidance of surgery and selection of conservative care. The clinical course and response patterns were not consistent with the initial working diagnosis, suggesting non-odontogenic pain. The final diagnoses were PIDP/PDAP (case 1), trigeminal neuralgia (case 3), TACs (case 4), and chronic myofascial pain (case 6). Provisional diagnoses included traumatic lingual nerve injury with neuropathic pain (case 2) and mixed neuropathic and myofascial pain (case 5).

Table 2 summarizes the individual courses. Fig. 1 depicts the diagnostic branching and pathways to correct the diagnosis.

• Median age of 74.5 years (interquartile range (IQR) 65–78); female 5/6.

• The median diagnostic delay (from symptom onset to the final or provisional diagnosis made in our department) was 17 months (IQR, 7–96 months).

• The median number of prior irreversible procedures (pulpectomy/extraction): 1 (range 0–2).

• The NRS score improved from a median of 8 at baseline to 0–2 at last observation. There was a ≥2-point improvement in all six (100%) cases. The median observation period in our department was 3.5 months (IQR, 3–14; range 1.4–19).

• Antibiotics and NSAIDs were administered as initial empiric dental management prior to referral in three and four patients, respectively (all failed to achieve remission, 100%).

• Autonomic signs (nasal congestion/conjunctival injection) were observed in one (17%) patient, who was ultimately diagnosed with TACs.

## 4. Discussion

Our findings indicate that, in the common clinical scenario of “treatment-refractory pain with negative dental findings”, suspending further irreversible procedures and systematically re-evaluating for non-odontogenic causes can be useful [7, 8, 9]. All but one of the patients were women (5/6; 83%). Although chronic orofacial pain is reported to be more prevalent in females [13], the present case series is too small to explore sex-related differences, and no statistical inference can be made.

The median diagnostic delay was 17 months, and the patients had a median of one prior irreversible procedure. Initial antibiotics (3/6) and NSAIDs (4/6) were ineffective. Contrastingly, the NRS score improved to 0–2 by last observation in all cases, which supports the clinical value of internalizing a “stop-and-re-evaluate” point for persistent pain that is poorly explained by inflammation/odontogenic frameworks.

### 4.1 Misdiagnosis factors and case-derived insights

Using this exploratory framework, four recurring domains of misdiagnosis were identified across the analyzed cases (Table 3). In particular, the misdiagnosis domains corresponded to: (1) overreliance on imaging and prior medication history, (2) overlooking autonomic signs, (3) insufficient musculoskeletal or neurological examination, and (4) premature attribution to a single etiology [7, 8, 9, 10, 11]. The three proposed red flags (stop points) applied to at least one item in every case, which suggested practical triggers for re-evaluation. However, given the small sample size (n = 6), their diagnostic performance requires prospective validation.

### 4.2 Cognitive biases potentially involved

Anchoring (fixation on an initial “odontogenic” hypothesis with underweighting of conflicting negatives), confirmation bias (selective search for supportive data), availability heuristic (overestimation of common odontogenic diseases), and overreliance on imaging were all suggested in our cases [7, 11, 17, 18]. Imaging should remain adjunctive and integrated with reproducible neurological and musculoskeletal findings.

### 4.3 Case-specific lessons

• Case 1: Persistent spontaneous pain with negative imaging/provocation following irreversible procedures should prompt re-evaluation of PIDP/PDAP [15, 19, 20].

• Case 2: Temporal linkage to dental extraction, unilateral lingual localization, and responsiveness to neuropathic agents all favored a diagnosis of traumatic lingual nerve injury [21, 22, 23] over chemotherapy-associated neuropathy [24]; however, the latter could not be fully excluded. Although the patient had a history of chemotherapy, their clinical presentation was more consistent with a localized traumatic trigeminal neuropathy than with chemotherapy-related neuropathy. The sensory disturbance was strictly confined to the lingual nerve distribution, unilateral, and demonstrated clear side-to-side differences. This sharply localized, single-branch trigeminal pattern is less typical for chemotherapy-related neuropathy, which more commonly presents as diffuse or multifocal sensory symptoms, rather than a focal cranial nerve distribution. Furthermore, symptom onset and persistence were temporally associated with the preceding dental extraction, further supporting a diagnosis of localized traumatic nerve injury, rather than systemic chemotherapy effects [24].

• Case 3: The patient’s initial diagnostic trajectory was strongly influenced by clinical context. She had a history of oral bisphosphonate use and underwent tooth extraction in February 2023, followed by additional curettage in May 2023. Pain remained consistently localized to the extraction site, and in the early disease stage, the patient described the pain as persistent, rather than paroxysmal. Given the presence of bisphosphonate exposure and ongoing post-extraction pain following repeated invasive procedures, MRONJ was initially considered the most plausible diagnosis, which was deemed clinically reasonable based on existing guidelines and risk stratification [25]. The predominance of invasive dental procedures and localized nociceptive input likely masked the underlying neural mechanism, as well as the absence of a clearly reported electric shock-like pain in the early phase, reduced the suspicion of trigeminal neuralgia. At the initial stage, pain assessment focused primarily on pain location and persistence, while key phenotypic features—such as attack duration, pain triggered by light mechanical stimuli, presence of brief paroxysms and trigger zones—were not systematically explored or documented. This limited the early recognition of any neural pain pattern. Upon systematic re-evaluation, critical features that had not been previously emphasized emerged, including brief paroxysmal attacks lasting seconds, electric shock-like quality, mechanical triggers during oral contact, and well-defined trigger zones. These findings fulfilled the diagnostic criteria for classical trigeminal neuralgia according to the ICHD-3 [3, 26], and were further supported by the rapid and sustained response to carbamazepine [27]. This case further illustrates a diagnostic pitfall in which anchoring to a high-risk medication history and local dental pathology may delay the consideration of trigeminal neuralgia, particularly when early pain descriptors are nonspecific and confined to the site of dental intervention.

• Case 4: Autonomic signs (nasal congestion and conjunctival injection) prompted early recognition and referral for TACs [2, 3, 16, 28].

• Case 5: Diagnosis and dominant mechanisms changed over time, which argued against premature closure and explicit on-hold diagnosis with iterative reassessment [7, 11, 17, 18]. Finally, comorbid neuropathic and myofascial pain was suspected [12, 29, 30].

• Case 6: Vascular contact on MRI alone should not determine trigeminal neuralgia [3, 26, 27]. Lack of classic paroxysms/trigger points and response to NSAIDs/muscle relaxants/rehabilitation are suggestive of myofascial pain [12, 29, 30]. However, a neuropathic contribution could not be fully excluded, given the long pain history and transient residual pressure-evoked tenderness despite resolution of spontaneous pain. This case illustrates the diagnostic complexity when neuropathic and musculoskeletal pain mechanisms may coexist [29].

### 4.4 Clinical significance of case 5: dynamic diagnostic reconstruction

Over a 6-year course, the diagnosis evolved from suspected PDAP to overt myofascial features and finally to comorbid neuropathic and myofascial pain. Treatment responses (tricyclics → carbamazepine → celecoxib) suggest shifting dominant mechanisms [13, 29]. Declaring “diagnosis pending”, with periodic re-evaluation and pharmacologic provocation as quasi-diagnostic probes, may improve accuracy [11, 17, 18]. For chronic orofacial pain, it is more realistic to seek functional improvement and acceptable pain, rather than complete remission [13, 30].

### 4.5 Pharmacotherapy: role and limits

Medication response informs, but should not dictate the diagnosis. For example, the considerable effect of carbamazepine in Case 3 supports (but does not solely establish) trigeminal neuralgia in conjunction with paroxysms and trigger points [3, 26, 27]. Pharmacological management should be guided by the final clinical diagnosis established using validated diagnostic criteria, rather than by the pain quality alone [2, 3, 12]. However, qualitative pain characteristics may provide supportive information during diagnostic reasoning [13]. For example, paroxysmal electric shock-like pain may raise the suspicion of trigeminal neuralgia [26, 27], while persistent burning or dysesthetic pain may indicate a neuropathic pain component [2, 13, 15]. Similarly, localized muscle tenderness or chewing-related pain may indicate a possible myofascial contribution [12, 30]. Importantly, these features were used only as adjunctive clues within a comprehensive diagnostic framework, not as standalone determinants of treatment selection.

Once a diagnosis has been established using validated criteria (ICOP, ICHD-3, DC/TMD), an evidence-based pharmacotherapy strategy can be selected accordingly [2, 3, 12]. For example, trigeminal neuralgia diagnosed per the ICHD-3 criteria is treated with carbamazepine as a first-line therapy [3, 26, 27]. Persistent idiopathic dentoalveolar pain meeting the ICOP criteria may be managed with gabapentinoids or tricyclic antidepressants based on evidence for neuropathic pain conditions [2, 13, 15, 19, 20]. Myofascial pain diagnosed per the DC/TMD criteria is treated with NSAIDs, muscle relaxants, and physical therapy [12, 29, 30]. Treatment response should inform, but not replace, systematic diagnostic evaluation, as multiple pain mechanisms may coexist (as demonstrated in Case 5) [29].

Sequential or combination pharmacotherapy may be necessary for cases of complex chronic pain [13]. However, in our practice, each medication trial was predefined with a fixed evaluation window (typically 2–4 weeks) and an a priori response threshold (≥2-point NRS improvement), to facilitate structured reassessment. Pharmacotherapy alone rarely yields complete remission, while rehabilitation and self-care are often essential [29, 30].

### 4.6 Implementing ”stop points” and a re-evaluation algorithm

The proposed “red flags” for diagnostic re-evaluation were primarily derived from the authors’ collective clinical experience in the management of treatment-refractory orofacial pain, and were informed by established textbooks and prior literature, rather than validated diagnostic criteria, and are illustrated in Fig. 1. Our two-stage exclusion (triage at referral and confirmation after the first visit) and independent dual-rater assignment with consensus may facilitate operationalization. Based on the ICOP/ICHD-3/DC/TMD criteria [2, 3, 12] and existing literature [7, 8, 9, 15], we propose three red flags:

1. Persistent pain objective negatives: No clear odontogenic pathology on imaging, no acute inflammatory signs, or negative provocation tests.

2. Atypical associated signs: ipsilateral nasal congestion, conjunctival injection, and tearing (autonomic signs).

3. Incongruent treatment response—insufficient response to usual dental management and/or atypical response patterns suggesting an alternative or mixed pain mechanism.

Conceptually, these red flags integrate the established diagnostic principles (ICOP for persistent dentoalveolar pain, ICHD-3 for trigeminal autonomic cephalalgias, and prior reports of treatment-refractory non-odontogenic pain) with our clinical observations [2, 3, 7, 8, 9, 15]. While the individual components have been well-described in the literature, their combination as a practical framework for triggering diagnostic re-evaluation represents our clinical synthesis and has not been prospectively validated.

In our six cases, there was at least one red flag per case. These indicators are hypothesis-generating and intended to function as clinical triggers prompting suspension of further irreversible dental procedures and the initiation of systematic diagnostic re-evaluation, rather than as standalone diagnostic tools. The presence of these factors should, therefore, prompt reassessment according to the ICOP/ICHD-3/DC/TMD criteria, including history, provocation testing, musculoskeletal and neurological examination, and evaluation of headache-associated signs [2, 3, 12]. Conversely, diagnoses of classic trigeminal neuralgia or TACs should prompt early specialty referral [3, 26, 27, 28].

### 4.7 Educational and clinical implications

Misdiagnosis and prolonged diagnostic delay in non-odontogenic orofacial pain impose substantial burdens on patients beyond physical symptoms. Qualitative studies have shown that patients with persistent orofacial pain frequently experience frustration, anxiety, erosion of trust in healthcare providers, and a sense of being dismissed or disbelieved when pain persists despite repeated interventions [31]. In our case series, the median diagnostic delay of 17 months and the repeated performance of irreversible dental procedures exemplify this problem. Diagnostic errors in medicine are often driven by cognitive biases, including anchoring and premature closure, which may perpetuate incorrect diagnostic trajectories once an initial diagnostic label has been applied [32].

The primary clinical implication of our study is the potential to reduce “diagnostic momentum”, where a patient is passed from one clinician to another with an incorrect label, leading to a cascade of unnecessary procedures. Our findings indicate that the proposed “stop-and-re-evaluate” protocol is not merely a specialist tool, but a fundamental safeguard in general dental practice. Implementing this pause when “red flags” are present could significantly minimize the physical burden of irreversible treatments (*e.g.*, pulpectomies and extractions) and the psychological distress associated with unresolved chronic pain. Furthermore, this study highlights the necessity of interdisciplinary collaboration. As observed in Cases 4 (TACs) and 6 (myofascial pain), the differential diagnosis commonly spans across the neurology and musculoskeletal domains. Therefore, the dental education curricula should place greater emphasis on the “medical” model of orofacial pain assessment, moving beyond the traditional “surgical/restorative” model. Establishing clear referral pathways between general dentists, oral surgeons, and neurologists is essential for the early management of conditions like TACs and trigeminal neuralgia.

### 4.8 Limitations and future directions

This study has several important limitations. First, as a single-center retrospective case series, the present study population was highly selected, consisting of referred patients following diagnostic difficulty or treatment failure. This referral pattern likely overrepresents complex or treatment-refractory cases with prolonged diagnostic delay, while underrepresenting patients who were successfully diagnosed and managed in general dental practice. Consequently, the distribution of diagnoses and diagnostic delay observed in this series is likely inapplicable to the broader dental population. Second, the retrospective design potentially introduced information bias, as clinical data were derived from medical records, referral letters, and patient recall, which varied in completeness and accuracy. This may have affected the assessment of symptom onset, prior treatments, and factors contributing to misdiagnosis. Third, comprehensive standardized QST using computerized equipment was not available in our clinical setting. Consequently, somatosensory evaluation relied on standardized chairside sensory testing, which may have limited full characterization of sensory profiles in patients with suspected neuropathic pain. Fourth, the proposed “red flags” represent hypothesis-generating indicators derived from the integration of clinical experience with established diagnostic concepts, rather than validated diagnostic criteria. Their diagnostic performance characteristics, including sensitivity, specificity, and predictive values, remain unknown and require prospective validation. Fifth, the small sample size (n = 6) and the heterogeneity of final diagnoses (persistent idiopathic dentoalveolar pain, traumatic neuropathy, trigeminal neuralgia, TACs, and myofascial pain) limited our ability to draw condition-specific conclusions. Furthermore, treatment approaches and follow-up durations varied across cases, precluding systematic evaluation of long-term outcomes. Accordingly, the findings of this case series should be interpreted as only hypothesis-generating. As such, future multicenter prospective studies with larger, unselected patient populations and standardized diagnostic protocols are warranted to validate the proposed clinical framework and red flags, to establish their diagnostic performance characteristics, and to assess their clinical utility in routine dental practice.

## 5. Conclusions

Overall, this case series suggests that early consideration of non-odontogenic causes is important in cases of orofacial pain unresponsive to usual dental treatment with objective negative findings (negative imaging, no acute inflammatory signs, and negative provocation tests). Factors contributing to misdiagnosis included overreliance on imaging or medication history, overlooking of autonomic signs, and premature attribution to a single cause, as described in the existing literature. Although treatment inefficacy itself was not a direct cause of misdiagnosis, non-response to initial therapy in the setting of objective negatives should be recognized as a key signal to trigger diagnostic re-evaluation. Suspending irreversible procedures and conducting systematic diagnostic reassessment within the established frameworks (ICOP, ICHD-3, and DC/TMD) is therefore a practical strategy. Our proposed stop points (red flags) should be regarded as hypothesis-generating clinical triggers, rather than diagnostic criteria and require prospective validation.
